# RURAL HEALTH AND FAMILY CAREGIVING: IDENTIFYING CHARACTERISTICS AND ADDRESSING NEEDS ACROSS POPULATIONS

**DOI:** 10.1093/geroni/igae098.2216

**Published:** 2024-12-31

**Authors:** Caroline Stephens, Jacqueline Telonidis, Katherine Ornstein

**Affiliations:** Chair; Co-Chair; Discussant

## Abstract

The rural population in the U.S. is growing and aging faster, and they have more comorbidities, than the overall aging population. Despite the greater need for resources and supports, rural residents may experience the most barriers to formal services (e.g., hospital, hospice/palliative care, nursing home care) and may lack available family caregivers. Unfortunately, there is a dearth of population-based research comparing the characteristics and needs of older adults living and dying in rural vs. urban areas, particularly related to availability of family. This symposium includes 4 presentations focused on rural health and family caregiving and identifying the characteristics and addressing needs across populations. The first presentation uses a retrospective population cohort design with cancer registry and state-wide claims data to examine urban/rural differences in palliative care-related service claims among older adult cancer patients diagnosed with metastatic breast, lung, colorectal, head and neck, bladder, and melanoma cancers. The next two presentations use data from the Utah Caregiving Population Study (Utah C-PopS) to examine how family size and characteristics impact EOL care utilization for urban vs. rural NH residents and for persons with dementia. The last presentation describes a program evaluation of an interagency collaboration to support caregivers in rural communities. Together, these presentations shed light on the diverse characteristics, gaps in care, and novel strategies to address the needs of rural populations to better inform rural health practice and policy considerations.

